# *De Novo* Cobalamin Biosynthesis, Transport, and Assimilation and Cobalamin-Mediated Regulation of Methionine Biosynthesis in Mycobacterium smegmatis

**DOI:** 10.1128/JB.00620-20

**Published:** 2021-03-08

**Authors:** Terry Kipkorir, Gabriel T. Mashabela, Timothy J. de Wet, Anastasia Koch, Lubbe Wiesner, Valerie Mizrahi, Digby F. Warner

**Affiliations:** a SAMRC/NHLS/UCT Molecular Mycobacteriology Research Unit, Department of Pathology and Institute of Infectious Disease and Molecular Medicine, Faculty of Health Sciences, DSI/NRF Centre of Excellence for Biomedical TB Research, University of Cape Town, Cape Town, South Africa; b Department of Integrative Biomedical Sciences, Faculty of Health Sciences, University of Cape Town, Cape Town, South Africa; c Division of Clinical Pharmacology, Department of Medicine, University of Cape Town, Cape Town, South Africa; d Wellcome Centre for Infectious Diseases Research in Africa, Faculty of Health Sciences, University of Cape Town, Cape Town, South Africa; Ohio State University

**Keywords:** vitamin B_12_, riboswitch, *cobK*, tuberculosis, *Mycobacterium tuberculosis*, mycobacterial metabolism

## Abstract

Cobalamin is an essential cofactor in all domains of life, yet its biosynthesis is restricted to some bacteria and archaea. Mycobacterium smegmatis, an environmental saprophyte frequently used as surrogate for the obligate human pathogen M. tuberculosis, carries approximately 30 genes predicted to be involved in *de novo* cobalamin biosynthesis. M. smegmatis also encodes multiple cobalamin-dependent enzymes, including MetH, a methionine synthase that catalyzes the final reaction in methionine biosynthesis. In addition to *metH*, M. smegmatis possesses a cobalamin-independent methionine synthase, *metE*, suggesting that enzyme use—MetH versus MetE—is regulated by cobalamin availability. Consistent with this notion, we previously described a cobalamin-sensing riboswitch controlling *metE* expression in M. tuberculosis. Here, we apply a targeted mass spectrometry-based approach to confirm *de novo* cobalamin biosynthesis in M. smegmatis during aerobic growth *in vitro*. We also demonstrate that M. smegmatis can transport and assimilate exogenous cyanocobalamin (CNCbl; also known as vitamin B_12_) and its precursor, dicyanocobinamide ([CN]_2_Cbi). However, the uptake of CNCbl and (CN)_2_Cbi in this organism is restricted and seems dependent on the conditional essentiality of the cobalamin-dependent methionine synthase. Using gene and protein expression analyses combined with single-cell growth kinetics and live-cell time-lapse microscopy, we show that transcription and translation of *metE* are strongly attenuated by endogenous cobalamin. These results support the inference that *metH* essentiality in M. smegmatis results from riboswitch-mediated repression of MetE expression. Moreover, differences observed in cobalamin-dependent metabolism between M. smegmatis and M. tuberculosis provide some insight into the selective pressures which might have shaped mycobacterial metabolism for pathogenicity.

**IMPORTANCE** Alterations in cobalamin-dependent metabolism have marked the evolution of Mycobacterium tuberculosis into a human pathogen. However, the role(s) of cobalamin in mycobacterial physiology remains poorly understood. Using the nonpathogenic saprophyte M. smegmatis, we investigated the production of cobalamin, transport and assimilation of cobalamin precursors, and the role of cobalamin in regulating methionine biosynthesis. We confirm constitutive *de novo* cobalamin biosynthesis in M. smegmatis, in contrast with M. tuberculosis, which appears to lack *de novo* cobalamin biosynthetic capacity. We also show that uptake of cyanocobalamin (vitamin B_12_) and its precursors is restricted in M. smegmatis, apparently depending on the cofactor requirements of the cobalamin-dependent methionine synthase. These observations establish M. smegmatis as an informative foil to elucidate key metabolic adaptations enabling mycobacterial pathogenicity.

## Abstract (precis)

Alterations in cobalamin-dependent metabolism have marked the evolution of Mycobacterium tuberculosis into a human pathogen. However, the role(s) of cobalamin in mycobacterial physiology remain poorly understood.

## INTRODUCTION

**FIG 1:**
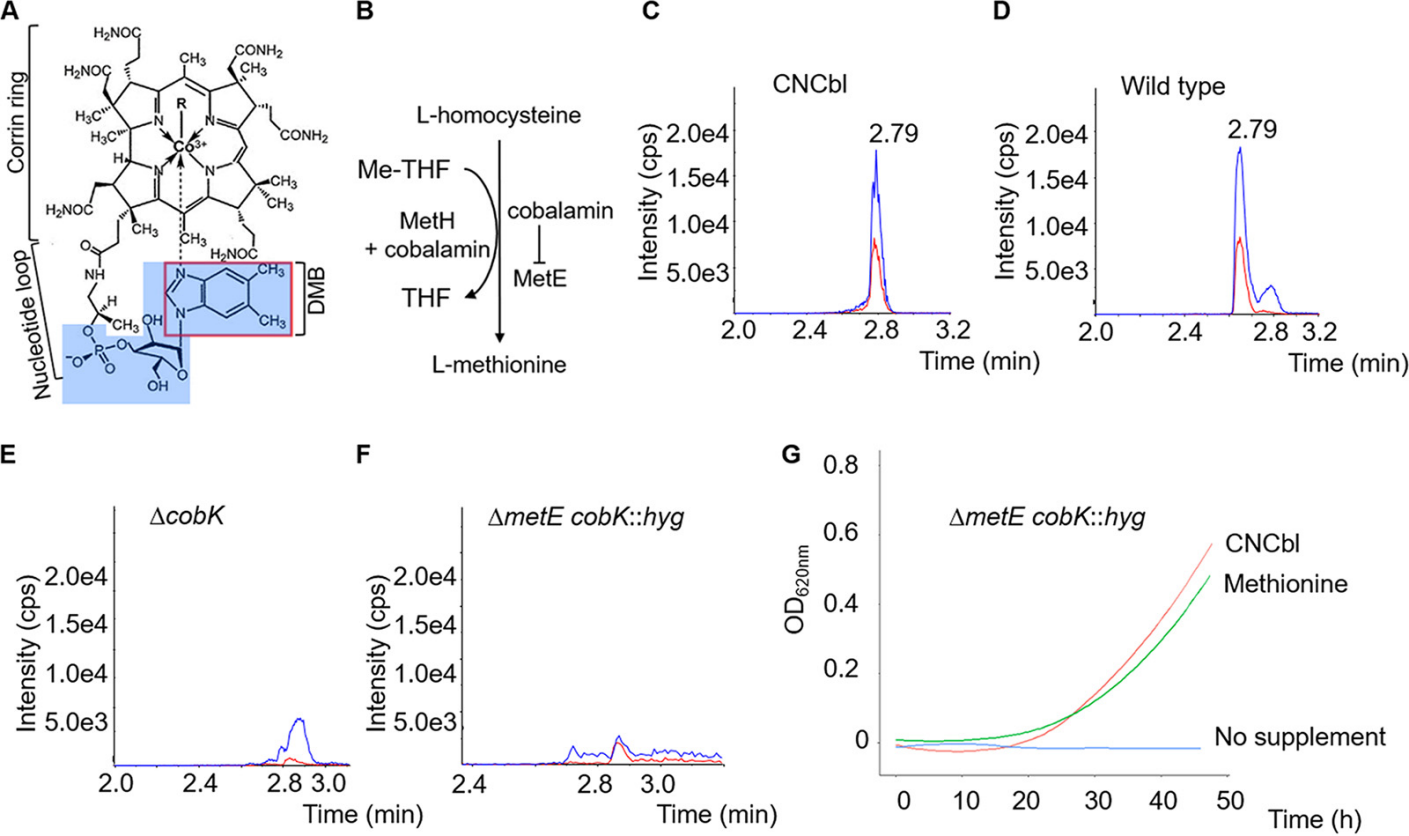
*De novo* cobalamin biosynthesis in M. smegmatis. (A) Cobalamin structure. The cobalt ion is coordinated equatorially by four nitrogen atoms of a corrin ring and axially by variable lower (α) and upper (β) ligands (R-group). Examples of β ligands are CN in cyanocobalamin (CNCbl; also known as vitamin B_12_), adenosyl in adenosylcobalamin (AdoCbl; also known as coenzyme B_12_), and methyl in methylcobalamin (MeCbl). The α ligand in the physiologically relevant cobalamin is typically dimethylbenzimidazole (DMB; outlined in red). (B) The final step in the methionine biosynthesis pathway is a nonreversible transfer of a methyl group from methyltetrahydrofolate (Me-THF) to homocysteine to produce methionine and tetrahydrofolate (THF). This reaction is catalyzed either by MetH using cobalamin as a cofactor or by MetE. MetE expression is attenuated by cobalamin via a cobalamin sensing riboswitch. (C) The LC-MS/MS method optimized to detect coeluting peaks corresponding to α-ribazole 5′-phosphate (highlighted in blue in panel A; blue trace in graphs) and DMB (red trace) transitions in a 20 ng/ml CNCbl standard. (D to F) Detection of *de novo* derivatized CNCbl. Cobalamin was detected in the wild type (D) but not in Δ*cobK* (E) and Δ*metE cobK*::*hyg* (F) mutants. The wild-type and Δ*cobK* strains were grown in 7H9-OADC medium and the Δ*metE cobK*::*hyg* strain was grown with 1 mM methionine supplementation. Peak intensities are expressed as counts per second (cps). (G) Growth curves of the Δ*metE cobK*::*hyg* strain in liquid 7H9-OADC medium in the presence of 10 μM CNCbl or 1 mM methionine. The mutant cannot grow without supplementation.

Several mycobacterial species have been identified among the subset of prokaryotes that possess the genetic capacity for *de novo* cobalamin biosynthesis (1–5). Included in this list of potential cobalamin producers is Mycobacterium smegmatis, the saprophytic mycobacterium commonly used experimentally as a surrogate for Mycobacterium tuberculosis, which causes tuberculosis (TB), a deadly respiratory disease claiming over 1 million lives globally every year (6–8). Cobalamin has one of the most complex structures of any of the biological cofactors, comprising a tetrapyrrole framework with a centrally chelated cobalt ion, dimethylbenzimidazole (DMB) as the lower axial base (α ligand), and an upper axial ligand (R-group; β ligand) (Fig. 1A). The nomenclature and catalytic activity of cobalamin depend on the β ligand. For example, in adenosylcobalamin (AdoCbl; also known as coenzyme B_12_), the β ligand is a deoxyadenosyl group utilized by isomerases such as the methylmalonyl coenzyme A (methylmalonyl-CoA) mutase and class II ribonucleotide reductases. In methylcobalamin (MeCbl), which serves as the substrate of methyltransferases such as methionine synthase, the β ligand is a methyl group (Fig. 1A) (9, 10).

Cobalamin biosynthesis is a complex multistep process requiring nearly 30 enzyme-catalyzed biotransformations, including eight *S*-adenosylmethionine (SAM)-dependent methylations, ring contraction, six amidations, decarboxylation, cobalt insertion, aminopropanol attachment, and the assembly and attachment of the α ligand (11). Owing to the heavy energetic investment necessary to support the *de novo* pathway, this process is typically augmented in many organisms by the capacity for uptake and salvage (1). Interestingly, cobalamin-producing bacteria encode a much larger complement of genes involved in biosynthesis and salvage than the number of cobalamin-dependent metabolic pathways in those organisms. The preservation of *de novo* biosynthetic capacity therefore suggests the contribution of cobalamin to adaptation to specific lifestyles—an interpretation which is especially intriguing in the context of pathogenic, parasitic, and symbiotic bacteria (4).

Like most mycobacteria, M. smegmatis encodes several cobalamin-dependent enzymes (Table 1) (12), some of which appear redundant given the existence of isoenzymes or alternative mechanisms for the same metabolic pathway (13). Among these are the methionine synthases, MetH (5-methyltetrahydrofolate-homocysteine methyltransferase, EC 2.1.1.14) and MetE (5-methyltetrahydropteroyltriglutamate-homocysteine methyltransferase, EC 2.1.1.13), which catalyze the nonreversible transfer of a methyl group from 5-methyltetrahydrofolate to homocysteine in the final step in the biosynthesis of methionine, an essential amino acid required for translation initiation, DNA methylation, and cysteine biosynthesis (14, 15). MetH requires cobalamin for activity, while MetE is a cobalamin-independent methionine synthase (Fig. 1B) (9, 16, 17). We previously demonstrated that a cobalamin-sensing riboswitch located in the 5′ untranslated region (5′ UTR) of the *metE* gene in M. tuberculosis attenuated *metE* transcript levels in the presence of exogenous cyanocobalamin (CNCbl; vitamin B_12_) (18). We also showed that M. tuberculosis CDC1551, a well-characterized isolate responsible for a TB outbreak in the United States (19), contains a natural truncation of the *metH* gene that renders the strain sensitive to exogenous CNCbl. This phenotype, which was recapitulated in an engineered M. tuberculosis H37Rv mutant containing an analogous *metH* truncation, suggested that the observed growth inhibition was due to methionine depletion resulting from the effective elimination of all methionine synthase activity in the CNCbl-replete environment (18).

**TABLE 1:** Predicted cobalamin-dependent enzymes in M. smegmatis

Gene	Annotation^*a*^	Cofactor	Reaction catalyzed
*MSMEG_3158* (*mutA*)	Methylmalonyl-CoA mutase, small subunit	AdoCbl	Isomerization
*MSMEG_3159* (*mutB*)	Methylmalonyl-CoA mutase large subunit	AdoCbl	
*MSMEG_0497*	Glycerol dehydratase large subunit	AdoCbl	Isomerization
*MSMEG_1547*	Glycerol dehydratase large subunit	AdoCbl	
*MSMEG_6321*	Glycerol dehydratase large subunit	AdoCbl	
*MSMEG_1553* (*eutB*)	Ethanolamine ammonia-lyase, large subunit	AdoCbl	Isomerization
*MSMEG_1554* (*eutC*)	Ethanolamine ammonia-lyase, light chain	AdoCbl	
*MSMEG_4185* (*metH*)	Methionine synthase	MeCbl	Methyl transfer

a Annotation is from reference 63.

We postulated that *metE* would similarly be subject to riboswitch-mediated repression in M. smegmatis, given the inferred genetic capacity for *de novo* cobalamin biosynthesis in this organism (5). Moreover, the cobalamin-mediated repression of *metE* would render *metH* essential for growth of M. smegmatis *in vitro*. In this study, we provide direct biochemical confirmation that M. smegmatis constitutively produces cobalamin *in vitro*. We further show that M. smegmatis utilizes exogenous CNCbl and dicyanocobinamide ([CN]_2_Cbi) as precursors for the biosynthesis of the physiologically relevant cobalamin cofactor. However, our results indicate that the uptake of these corrinoid precursors by M. smegmatis is restricted. Finally, we demonstrate that the expression of *metE* in M. smegmatis is under constitutive repression by a cobalamin riboswitch, a finding that explains the essentiality of *metH* in this nonpathogenic mycobacterium under standard culture conditions.

## RESULTS

### *De novo* cobalamin biosynthesis pathway is functional in M. smegmatis.

Genomic analyses indicate that M. smegmatis encodes the complete pathway for *de novo* cobalamin biosynthesis (5). To investigate the ability of M. smegmatis to synthesize cobalamin using the *de novo* pathway, we developed a liquid chromatography-tandem mass spectrometry (LC-MS/MS) method based on the derivatization of cobalamin to CNCbl by potassium cyanide (KCN). Then, utilizing multiple reaction monitoring (MRM) of two coeluting transitions corresponding to α-ribazole 5′-phosphate and DMB (Fig. 1A), we identified cobalamin in the cell extracts as derivatized CNCbl, validated by two transitions coeluting at 2.79 min (Fig. 1C). Using this method, high-intensity CNCbl peaks were identified in cell extracts of wild-type M. smegmatis strain mc^2^155 grown aerobically to stationary phase in standard Middlebrook 7H9–oleic acid-albumin-dextrose-catalase (OADC) medium (Fig. 1D). To confirm *de novo* cobalamin production in M. smegmatis, we generated an unmarked, in-frame deletion of *cobK* (see Fig. S1A and C to E in the supplemental material). Homologs of this gene, which encodes a putative precorrin-6A reductase (5, 11, 20), have been shown in other organisms to be required for corrin ring synthesis (21–23). To ensure that all phenotypes reflected the consequences of the specific gene deletions and/or disruptions and were not confounded by off-site mutations, the parental strain and derivative mutants were subjected to whole-genome sequencing. Single nucleotide mutations (SNMs) in six genes were uniquely identified in the Δ*cobK* strain but not in the parental wild-type strain, but none of the SNMs could be linked to cobalamin or methionine metabolic pathways (Table 2).

**TABLE 2:** SNMs unique to the Δ*cobK* strain relative to the wild-type parental strain

Gene	Annotation^*a*^	SNM (genome position)^*b*^	Type of SNM^*b*^	Amino acid change
*MSMEG_0691*	Putative transcriptional regulatory protein	T>G (782521)	5′ UTR mutation	
*MSMEG_2148*	HNH endonuclease domain protein	T>C (2223876)	Missense	Trp379Arg
*MSMEG_3876*	Putative phosphotransferase enzyme family protein	G>A (3948881)	Missense	Arg296His
*MSMEG_6127*	Anti-anti-sigma factor	T>G (6190150)	Missense	Leu36Arg
*MSMEG_6270*	Hypothetical protein	A>C (6336541)	5′ UTR mutation	
		G>A (6336542)	5′ UTR mutation	
*MSMEG_6423*	Glycerophosphoryl diester phosphodiesterase family protein	T>C (6497015)	5′ UTR mutation	
		T>G (6497036)	5′ UTR mutation	

a Annotation is from reference 63.

b SNM, single nucleotide mutation; SNP, single nucleotide polymorphism; UTR, untranscribed region.

In contrast to wild-type cell extracts, the Δ*cobK* extracts lacked the dual coeluting peaks characteristic of CNCbl (Fig. 1E). This observation indicated the indispensability of CobK for cobalamin biosynthesis and provided further evidence that the cobalamin signal detected in the wild-type strain (Fig. 1D) resulted from *de novo* biosynthesis. We also confirmed the absence of cobalamin production in a double Δ*metE cobK*::*hyg* knockout (KO) strain during growth in l-methionine-supplemented medium (Fig. 1F). This strain, in which the entire *metE* open reading frame (ORF) is deleted and *cobK* is disrupted by the insertion of a hygromycin (*hyg*) resistance marker (Fig. S1B and F), is a methionine auxotroph that can be propagated only in media supplemented with methionine or CNCbl (Fig. 1G).

### M. smegmatis assimilates exogenous CNCbl and (CN)_2_Cbi *in vitro*.

**FIG 2:**
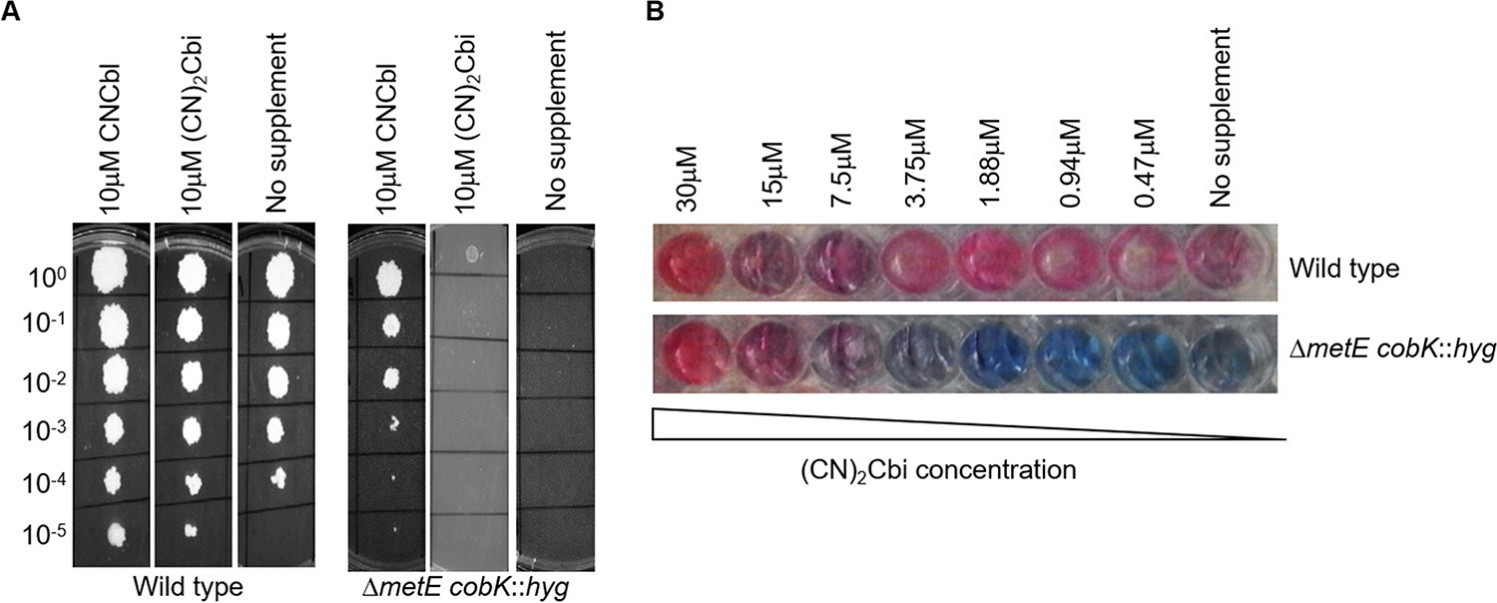
Uptake of exogenous CNCbl and (CN)_2_Cbi in M. smegmatis. (A) Spotting assays of exponential-phase cultures of wild-type and Δ*metE cobK*::*hyg* strains on 7H10-OADC agar with or without 10 μM CNCbl or 10 μM (CN)_2_Cbi show restricted uptake of (CN)_2_Cbi relative to CNCbl uptake on solid medium. (B) alamarBlue assay to evaluate the growth of the Δ*metE cobK*::*hyg* strain in liquid medium supplemented with (CN)_2_Cbi. Cells (5 × 10^3^) were seeded in 7H9-OADC medium supplemented with 2-fold dilutions of (CN)_2_Cbi starting at 30 μM as the highest concentration.

The ability to propagate the Δ*metE cobK*::*hyg* mutant in media containing methionine or CNCbl indicated that M. smegmatis can utilize exogenous methionine and CNCbl in the absence of an intact *de novo* cobalamin biosynthesis pathway, pointing to functional transport and assimilation pathways. We previously showed that M. tuberculosis could utilize dicyanocobinamide ([CN]_2_Cbi) during growth *in vitro* (12). To determine whether M. smegmatis could also assimilate this cobalamin precursor, we tested the ability of the Δ*metE cobK*::*hyg* double mutant to grow in medium supplemented with (CN)_2_Cbi. First, the strain was grown to exponential phase with excess methionine (1 mM), after which 10-fold serial dilutions were spotted onto Middlebrook 7H10-OADC agar containing 10 μM (CN)_2_Cbi. After a 3-day incubation at 37°C, (CN)_2_Cbi uptake was qualitatively assessed by examining colony sizes (Fig. 2A). For comparison, serial dilutions were also spotted on agar supplemented with 10 μM CNCbl. Interestingly, the growth of the Δ*metE cobK*::*hyg* strain was very limited on agar supplemented with (CN)_2_Cbi (Fig. 2A). In fact, growth was observed only in the most concentrated (undiluted) bacterial spots (Fig. 2A). While this might have been as a consequence of methionine carryover, the fact that similar growth was not observed in the unsupplemented 7H10 plate (Fig. 2A) suggested this was not the case. Instead, these observations implied the ability of M. smegmatis to utilize (CN)_2_Cbi, albeit to a much lesser extent than CNCbl (Fig. 2A). To test the potential for (CN)_2_Cbi to support growth in liquid culture, an “MIC-type” alamarBlue assay (24) was performed in which growth from an inoculum of ∼5 × 10^3^ Δ*metE cobK*::*hyg* cells was determined in medium containing 2-fold serial dilutions of (CN)_2_Cbi (Fig. 2B). The Δ*metE cobK*::*hyg* strain was viable at (CN)_2_Cbi concentrations higher than 7.5 μM (Fig. 2B), consistent with the ability to convert the corrinoid precursor to cobalamin.

**FIG 3:**
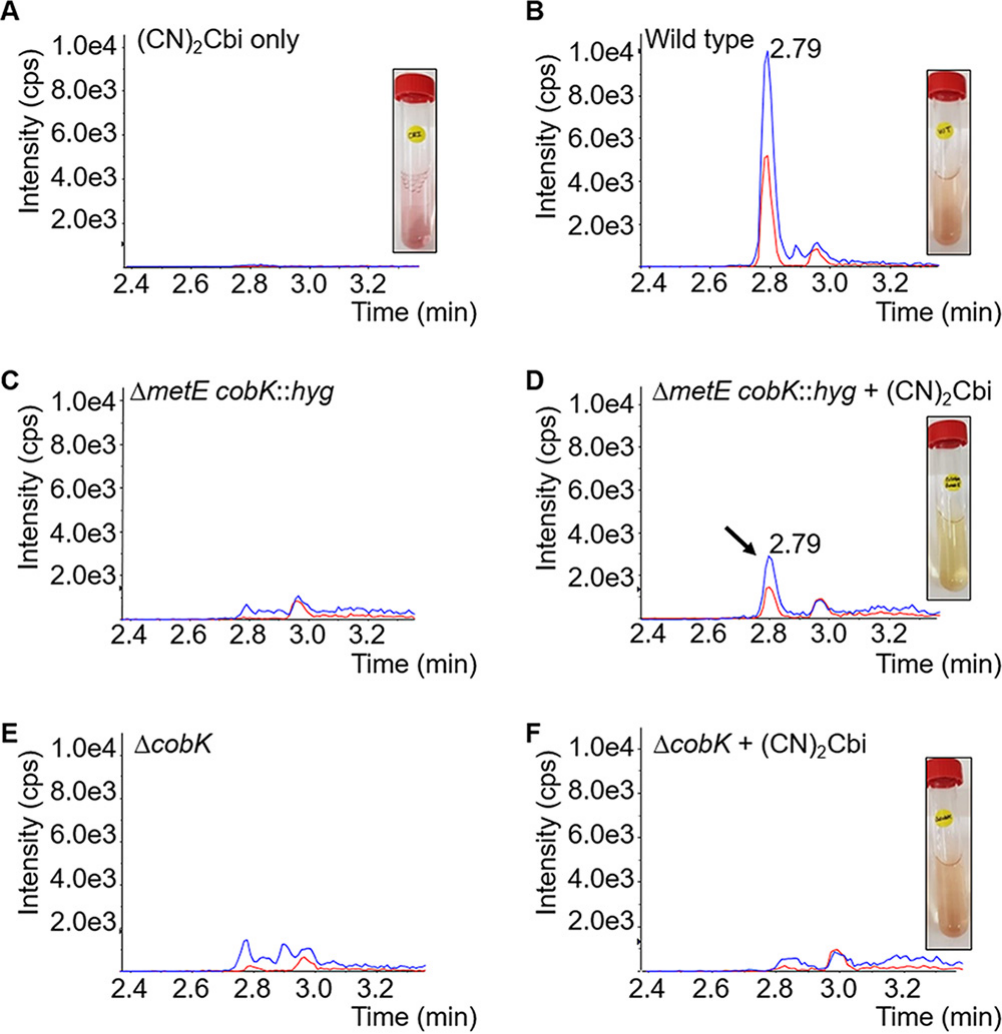
Assimilation of (CN)_2_Cbi in M. smegmatis. (A) (CN)_2_Cbi only control. (B) *De novo*-synthesized cobalamin in the wild-type strain. (C and D) Detection of recovered cobalamin due to (CN)_2_Cbi assimilation in the Δ*metE* Δ*cobK*::*hyg* double mutant. (E and F). Absence of recovered cobalamin in the Δ*cobK* strain in the presence of exogenous (CN)_2_Cbi. (CN)_2_Cbi uptake was accompanied by changes in the color of the spent medium from purple (A, inset) to a rusty hue in the wild-type (B, inset) and Δ*cobK* strains (F, inset), and pale yellow in the Δ*metE* Δ*cobK*::*hyg* strain (D, inset). Supplemented cultures contained 30 μM (CN)_2_Cbi.

To confirm the assimilation of (CN)_2_Cbi in M. smegmatis, we used LC-MS/MS to analyze cell extracts of wild-type, Δ*cobK*, and Δ*metE cobK*::*hyg* strains grown to stationary phase in 7H9-OADC medium with or without excess (30 μM) (CN)_2_Cbi (Fig. 3). As a positive control for *de novo* cobalamin biosynthesis, we analyzed the wild-type strain grown in parallel without supplementation (Fig. 3B). For the Δ*metE cobK*::*hyg* mutant, methionine supplementation was used to enable propagation in the absence of (CN)_2_Cbi (Fig. 3C). We observed that all the (CN)_2_Cbi-supplemented strains reached stationary phase simultaneously. However, the dual coeluting peaks characteristic of CNCbl were detected only in the (CN)_2_Cbi-supplemented Δ*metE cobK*::*hyg* strain (Fig. 3C and D), strongly suggesting uptake and conversion of (CN)_2_Cbi to cobalamin. Interestingly, cobalamin was not detectable in the (CN)_2_Cbi-supplemented Δ*cobK* strain (Fig. 3E and F), which did not require supplementation for growth. The assimilation of (CN)_2_Cbi in the Δ*metE cobK*::*hyg* strain was also accompanied by a distinct change in the color of the spent medium from purple to pale yellow (Fig. 3D, inset). By comparison, the color of the spent medium in the (CN)_2_Cbi-supplemented wild-type and Δ*cobK* cultures changed only slightly to a rusty hue (Fig. 3B and F, insets), consistent with limited uptake in these strains.

### MetE expression in M. smegmatis is regulated by a cobalamin-sensing riboswitch.

**FIG 4:**
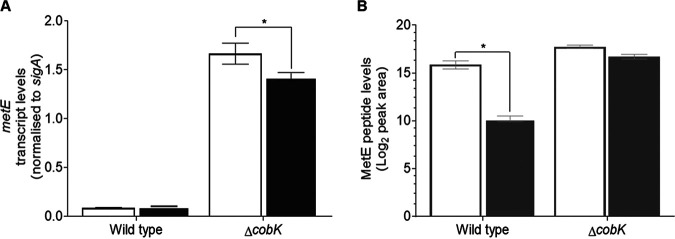
Cobalamin-mediated attenuation of MetE expression in M. smegmatis. (A) ddPCR analysis of *metE* transcription during exponential growth phase in the wild-type and Δ*cobK* strains cultured in the presence (solid bars) or absence (open bars) of exogenous CNCbl. The Δ*cobK* strain exhibited an overabundance of *metE* transcripts relative to the wild-type parental strain. A small but statistically significant decrease in the level of *metE* transcript in the Δ*cobK* strain was observed in the presence of exogenous CNCbl (two-way ANOVA; *, *P = *0.0359), but the change in *metE* transcript levels in the wild-type strain was not statistically significant. The graphed data are representative of two independent experiments. Error bars show the standard error of the mean. (B) Targeted MS analysis of MetE peptide levels (log_2_ peak area) in the wild-type and Δ*cobK* strains grown in the presence (solid bars) or absence (open bars) of exogenous CNCbl. Exogenous CNCbl more significantly decreased MetE peptide levels in the wild-type strain relative to the Δ*cobK* mutant (two-way ANOVA; *, *P = *0.0151).

We previously reported that the cobalamin-sensing riboswitch located in the 5′ UTR of *metE* strongly attenuated the transcription of this gene in M. tuberculosis in the presence of exogenous CNCbl (18). To investigate whether the corresponding riboswitch in M. smegmatis operated similarly, we analyzed relative *metE* transcript levels during the exponential-growth phase in wild-type and Δ*cobK* strains grown in the presence or absence of CNCbl using droplet digital PCR (ddPCR). We found low but detectable levels of *metE* transcripts in the wild-type strain (Fig. 4A). By comparison, *metE* transcripts were 19× more abundant in the Δ*cobK* strain (Fig. 4A), supporting the notion that the abrogation of *de novo* cobalamin biosynthesis in the mutant released *metE* transcription from riboswitch-mediated repression. There was no significant difference in *metE* transcript levels between the CNCbl-supplemented and unsupplemented wild-type strain (Fig. 4A). In contrast, a small but statistically significant reduction (0.86×) in *metE* transcript levels was observed in the Δ*cobK* strain in the presence of exogenous CNCbl (Fig. 4A). The absent to modest change in *metE* levels in these strains following CNCbl supplementation suggested that the uptake/assimilation of exogenous CNCbl might be restricted in M. smegmatis, consistent with the LC-MS/MS results. Alternatively, these results could indicate selective repression of cobalamin uptake/assimilation systems in strains which do not require the cofactor for viability or growth.

To examine how cobalamin availability in M. smegmatis affected MetE protein content, we adapted a targeted MS method (25) to measure MetE peptide levels in wild-type and Δ*cobK* strains grown in the presence or absence of exogenous CNCbl. This analysis indicated that MetE protein levels were 3-fold more abundant in the Δ*cobK* mutant than in the parental wild-type strain (Fig. 4B). In the presence of exogenous CNCbl, the Δ*cobK* mutant exhibited a 2-fold decrease in MetE protein levels (Fig. 4B). Unexpectedly, exposure of the wild-type strain to exogenous CNCbl caused a 48-fold reduction in MetE protein levels (Fig. 4B). This result contrasted with the modest impact of CNCbl on *metE* transcript levels (Fig. 4A) and suggested that MetE expression was likely primarily controlled at the translational level by this riboswitch.

### *metH* is essential in M. smegmatis.

The inferred cobalamin-mediated repression of *metE* in turn implied that MetH function would be indispensable for the growth of wild-type M. smegmatis. To test this prediction, we attempted to generate an in-frame *metH* deletion mutant (Fig. S1A) by two-step allelic exchange mutagenesis (26). The Δ*metH* construct was designed to mimic a naturally occurring *metH* truncation which partially disrupts the cobalamin-binding domain and eliminates the *S*-adenosyl-l-methionine (SAM)-binding domain of MetH in M. tuberculosis strain CDC1551 (18). Another *metH* KO construct containing a *hyg* marker (Fig. S1A) was also designed to enable the recovery of *metH* double crossover (DCO) mutants by “forced” selection on Hyg. Anticipating the loss of viability owing to *metH* essentiality, all media were supplemented with 1 mM l-methionine. Of 154 putative *metH* DCO recombinants screened by PCR, none (0/154) carried the Δ*metH* allele; instead, all 154 colonies were wild-type revertants. Similarly, 60 putative *hyg*-marked DCOs were screened by PCR, none of which bore the Δ*metH* allele. These results strongly suggested that *metH* was essential in M. smegmatis, consistent with recent genetic screens which identified *metH* among the subset of essential genes in M. smegmatis (27, 28).

### Conditional depletion by CRISPRi confirms *metH* essentiality.

**FIG 5:**
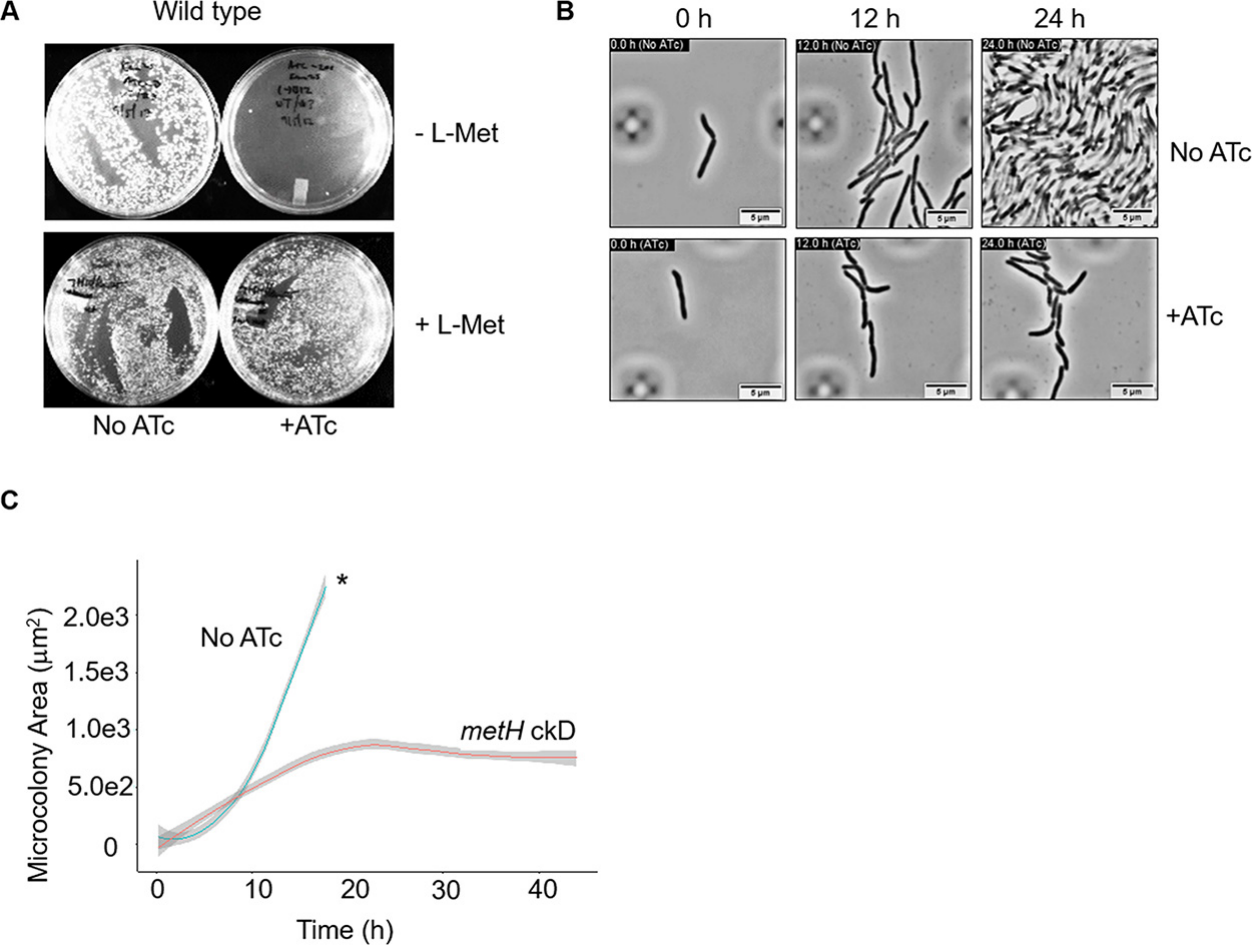
Growth cessation due to methionine depletion in the *metH* cKD strain. (A) ATc-induced growth inhibition in the *metH* cKD strain is rescued by exogenous methionine (l-Met). (B) Representative images from time-lapse microscopy at the 0-h, 12-h, and 24-h time points showing severe growth retardation in the *metH* cKD strain. Images are taken from Movie S2, available at https://uct.figshare.com/s/65105b9914196c4b4654. Scale bars, 5 μm. (C) Quantification of microcolony growth in the M. smegmatis *metH* cKD strain using “R” software; *, limit of detection.

Since our attempts to delete *metH* in M. smegmatis were unsuccessful, we instead opted to generate a *metH* conditional knockdown (cKD). For this purpose, we employed the anhydrotetracycline (ATc)-inducible mycobacterial CRISPRi system (29), utilizing a panel of 13 short guide RNAs (sgRNAs) targeting different regions of the *metH* ORF and with different target complementarity scores (Table S3). An sgRNA targeting *mmpL3*, an essential gene involved in mycolic acid biosynthesis (30), was used as a positive control. Gene silencing in transformed cells was assessed by growth inhibition on ATc-containing selection medium. The induction of *metH* silencing by ATc inhibited colony formation in wild-type M. smegmatis, confirming the essentiality of *metH* under the conditions tested (Fig. 5A). Consistent with previous work (28, 29, 31), sgRNAs with higher complementarity scores displayed stronger gene silencing, leading to more robust growth inhibition than sgRNAs with lower scores (Fig. S2). ATc-dependent growth inhibition was rescued by supplementation with exogenous methionine (Fig. 5A), indicating that the lack of growth in the *metH* cKD strains resulted from methionine starvation.

To investigate this phenotype further, we traced the growth of the *metH* cKD strain at the single-cell level using microfluidics and time-lapse microscopy. A log-phase culture of cells carrying the *metH* cKD construct was preincubated with ATc for 6 h at 37°C and then loaded into the CellASIC ONIX2 microfluidic device and imaged in real-time over the course of 43 h with constant perfusion with 7H9-OADC medium containing ATc. In parallel, an uninduced (no-ATc) control was perfused with 7H9-OADC medium only. Analysis of the time-lapse images showed that the induced *metH* cKD strain shared similar morphological features of shape and size with the no-ATc control (Fig. 5B). Moreover, in both the *metH* cKD strain and the no-ATc control, cells divided by v-snapping (Movies S1 to S5, available at https://uct.figshare.com/s/65105b9914196c4b4654), which is typical of mycobacterial cell division (32, 33). However, the growth rates of the *metH* cKD strain and the no-ATc control were markedly different (Fig. 5C). In the no-ATc control, microcolonies displayed an exponential-phase doubling time of 2.94 ± 0.3 h (Fig. 5C). In this control, tracing of distinct cells was feasible only for the first 18 h of the experiment; by 24 h, microcolonies had attained confluence, occupying the entire field of view (Fig. 5B; Movies S1 to S5 at the URL mentioned above). Consistent with methionine depletion in the *metH* cKD strain, this mutant exhibited a much slower replication rate, doubling every 5.23 ± 0.4 h until the 18-h time point when the growth rate flatlined (Fig. 5C) and the expansion of microcolonies slowed and appeared to halt by 24 h (Fig. 5B; Movies S1 to S5 at the URL mentioned above).

### Abrogation of cobalamin biosynthetic capacity alleviates *metH* essentiality in M. smegmatis.

**FIG 6:**
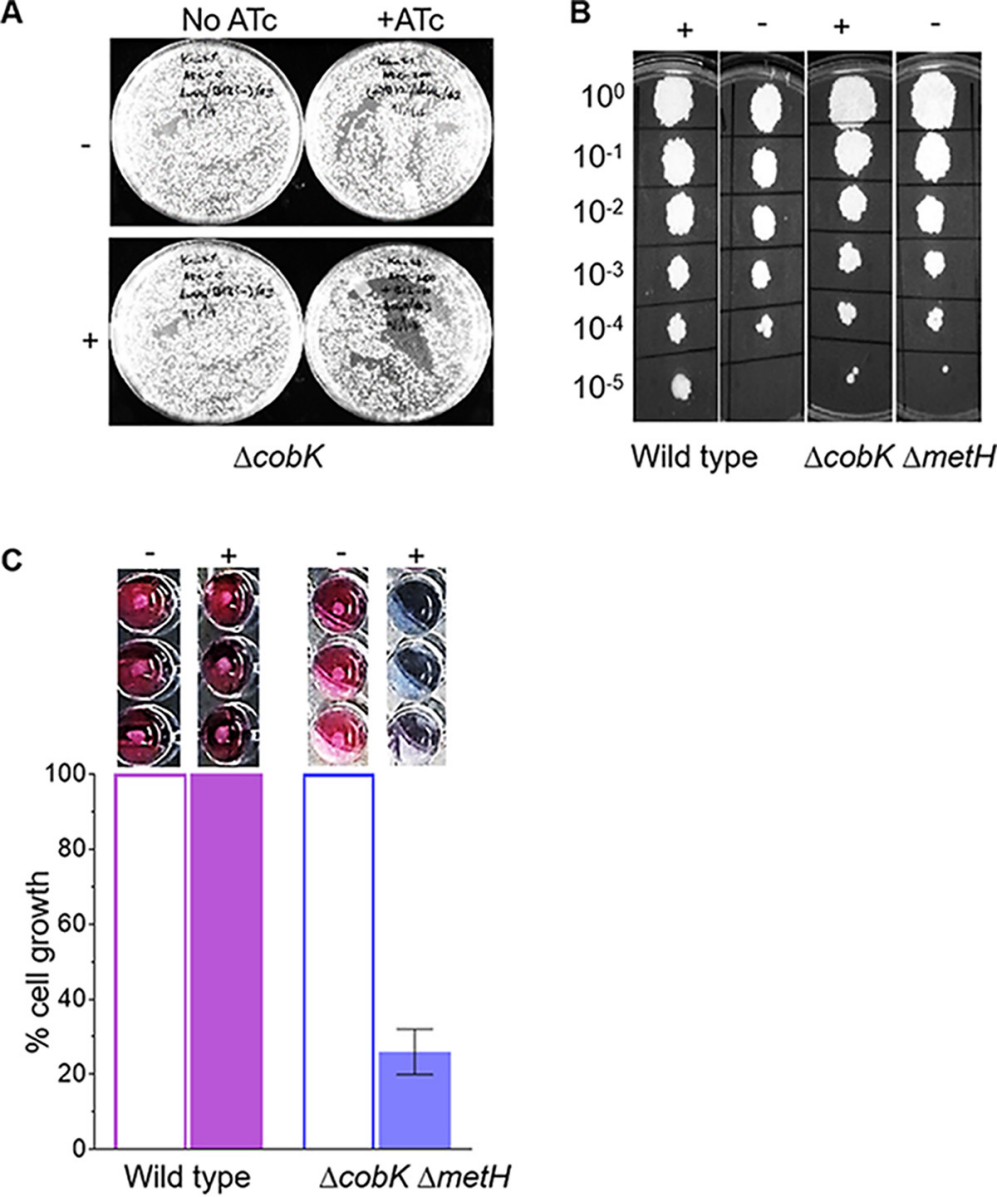
Sensitivity of MetH mutants to exogenous CNCbl. (A) CRISPRi-mediated silencing of *metH* in the Δ*cobK* strain in the presence (+) or absence (−) of exogenous CNCbl. Exogenous CNCbl had negligible effect on the growth of the *metH* cKD strain on solid medium in this background. (B) Spotting assay on 7H10 agar containing 10 μM exogenous CNCbl showing the failure of exogenous CNCbl to inhibit growth of the Δ*cobK* Δ*metH* strain on solid medium. (C) Sensitivity of the Δ*cobK* Δ*metH* strain to exogenous CNCbl in liquid medium, as determined using the alamarBlue assay.

The time-lapse microscopy data suggested that the essentiality of the *metH* gene might depend on both the presence of endogenous cobalamin and a functional *metE* riboswitch in M. smegmatis. Therefore, we reasoned it would be possible to create a *metH* deletion in the cobalamin-deficient Δ*cobK* strain. To test this hypothesis, we generated an unmarked in-frame *metH* deletion in the Δ*cobK* background and screened the resultant DCOs by PCR. As expected, PCR screening identified 9 out of 34 putative DCOs as Δ*cobK* Δ*metH* double KO mutants (Fig. S1C to E), linking the essentiality of *metH* to endogenous cobalamin availability. We found that silencing of *metH* had no effect on the viability of the cobalamin-deficient Δ*cobK* mutant (Fig. 6A), confirming that endogenous cobalamin was required to block methionine biosynthesis via riboswitch-mediated repression of *metE*. Moreover, consistent with the limited impact of exogenous CNCbl on MetE protein levels (Fig. 4B), CNCbl supplementation had a negligible effect during the growth of the Δ*cobK* strain on solid medium following ATc-induced *metH* silencing (Fig. 6A).

Next, we investigated the impact of exogenous CNCbl during growth of the Δ*cobK* Δ*metH* strain on solid versus liquid medium. To this end, a late log-phase (optical density at 600 nm [OD_600_] ∼1) culture was 10-fold serially diluted and spotted onto 7H10-OADC agar supplemented with or without 10 μM CNCbl (Fig. 6B). There was no impairment of growth of the CNCbl-supplemented Δ*cobK* Δ*metH* mutant on solid medium (Fig. 6B). To determine if this phenotype was also observed in liquid medium, we seeded an inoculum of 2.5 × 10^3^ Δ*cobK* Δ*metH* cells and analyzed cell proliferation after an overnight incubation at 37°C with or without 10 μM CNCbl using the alamarBlue assay (24) (Fig. 6C). Interestingly, CNCbl supplementation led to approximately 80% inhibition of the growth of the Δ*cobK* Δ*metH* strain (Fig. 6C). This result confirmed the ability of M. smegmatis to assimilate exogenous CNCbl, although the uptake of the corrinoid was seemingly better in liquid than on solid medium.

## DISCUSSION

### Cobalamin production in M. smegmatis.

The production of cobalamin by M. smegmatis was previously inferred indirectly from microbiological assays (34–36). Using a targeted LC-MS/MS approach, we provide direct proof of constitutive *de novo* cobalamin biosynthesis in M. smegmatis under aerobic conditions. The LC-MS/MS method optimized in this work utilized MRM of two mass spectra corresponding to DMB, the lower base in physiologically relevant cobalamin. Hence, we infer that M. smegmatis is able to synthesize and use DMB. Cobamides known as “pseudocoenzyme B_12_,” comprising an adenosine group as the α-axial ligand, rather than DMB, have been found in other bacteria (37, 38). M. smegmatis CobT is homologous to the corresponding proteins in Sinorhizobium meliloti and Salmonella enterica, which have been implicated in the incorporation of adenine as the α-ligand of pseudocoenzyme B_12_ under limited DMB availability (38). It is conceivable, therefore, that M. smegmatis might possess similar capacity for pseudocoenzyme B_12_ synthesis. Since this remains unexplored, we cannot exclude the possibility that our DMB-dependent LC-MS/MS detection method did not capture the full variety of cobamides present in M. smegmatis. Nonetheless, these results indicated substantial cobalamin production in M. smegmatis, consistent with an early study which detected low-level cobalamin production in M. smegmatis using the Lactobacillus leichmannii tube assay (34). The disruption of *cobK*, encoding a predicted precorrin-6A reductase, abrogated cobalamin production, confirming that a single genetic lesion can cripple the entire pathway. It is noteworthy, therefore, that the loss of *cobF*, encoding a putative precorrin-6A synthase occurring immediately upstream of CobK in the pathway, has been identified as one of the defining molecular events in the evolution of pathogenic mycobacteria (39–41).

### Corrinoid transport in M. smegmatis.

Both CNCbl and (CN)_2_Cbi supported the growth of the Δ*metE cobK*::*hyg* strain by enabling MetH-dependent production of methionine. These results demonstrate that M. smegmatis is capable of corrinoid transport and assimilation. However, our observation of strikingly restricted corrinoid transport in M. smegmatis raises questions about the corrinoid concentrations encountered in its natural habitats, and suggests the unlikelihood that exogenous corrinoids constitute a reliable source. CNCbl and (CN)_2_Cbi must undergo decyanation and adenosylation to produce adenosylcobalamin. While it is possible that M. smegmatis encodes enzymes for these reactions, work in other bacteria has demonstrated that decyanation can occur via the reduction of Co(III) to Co(II) without the need of specific decyanases (42). It has further been proposed that the steps involved in the reduction of Co(III) to Co(I), which is required for adenosylation, are not driven by reductases but, rather, are likely facilitated by electron transfer proteins (43). It seems probable, therefore, that bioconversion of CNCbl and (CN)_2_Cbi in M. smegmatis similarly occurs without the need for specific enzymes.

Interestingly, our data showed a seemingly enhanced capacity for (CN)_2_Cbi assimilation in the Δ*metE cobK*::*hyg* strain compared to wild-type and Δ*cobK* strains. Since both these strains are able to grow without cobalamin owing to the presence of MetE as alternative methionine synthase, the reduced (CN)_2_Cbi uptake might suggest the potential for selective assimilation/transport as a function of methionine biosynthetic capacity. The peak intensity of the recovered cobalamin in the (CN)_2_Cbi-supplemented Δ*metE cobK*::*hyg* strain was significantly lower than that of *de novo*-synthesized cobalamin in the wild type (Fig. 3B and D), possibly indicating that much smaller amounts of cofactor are necessary to support growth.

In M. tuberculosis, the nonspecific ABC-type transporter BacA (Rv1819c) has been identified as the sole cobalamin and corrinoid transporter (12). Until recently, the mechanistic details of cobalamin transport by Rv1819c had remained elusive. However, the resolution of the crystal structure of Rv1819c (44) has provided key insights into its function in the uptake of hydrophilic molecules, suggesting that this protein passes a cargo slowly along its cavity via facilitated diffusion. Facilitated diffusion is a very low-efficiency process and, if the M. smegmatis homologue functions similarly in corrinoid uptake, it might explain the poor uptake of CNCbl and (CN)_2_Cbi. M. smegmatis also contains two predicted Rv1819c homologues, encoded by paralogous genes located at different genomic loci (MSMEG_3655 and MSMEG 4380). In addition, M. smegmatis contains an operon encoding putative homologues of BtuF (MSMEG_4560), BtuC (MSMEG_4559), and BtuD (MSMEG_4558), all components of the classic TonB-ExBD-BtuFCD cobalamin transport system in Gram-negative bacteria (45). Whether these genes encode functional transporters is still unknown and further research is needed to determine which proteins are involved in ferrying corrinoids and their precursors across the notoriously complex mycobacterial cell wall (46).

### A riboswitch controls *metE* expression in M. smegmatis.

We previously reported that a cobalamin-sensing riboswitch controlled *metE* transcription in M. tuberculosis (18). In that work, the level of *metE* transcript was decreased in the presence of exogenous CNCbl, leading to the conclusion that this riboswitch functioned as a transcriptional “off” switch. In the current study, we found that the levels of *metE* transcript were much lower in the cobalamin-replete wild-type M. smegmatis strain compared to the Δ*cobK* mutant. Since riboswitches sense ligand levels to attenuate expression (47), the low-level *metE* transcripts found in the wild-type strain likely reflects a physiological equilibrium between ligand-bound and unbound riboswitch states, which allows for limited gene expression. Therefore, although the uptake of exogenous CNCbl is restricted in both wild-type and Δ*cobK* strains, the low level of uptake was still enough to shift the endogenous ligand-riboswitch equilibrium more significantly in wild-type than in mutant cells, which exhibited elevated MetE protein content (Fig. 4). Unlike in M. tuberculosis, exogenous CNCbl was unable to exert significant changes to *metE* transcript levels in M. smegmatis, presumably owing to the limited uptake. While these results imply transcriptional regulation, we also observed an unexpected and dramatic reduction in MetE protein levels in the wild-type strain in the presence of exogenous CNCbl. These results suggested that this riboswitch might utilize a coupled translational-transcriptional regulation mechanism by which the inhibition of translation initiation precedes transcription termination and mRNA instability (48–50). Future work will elucidate the precise mechanism of cobalamin-sensing riboswitches in mycobacteria.

### Lack of MetH activity is detrimental to the growth of M. smegmatis.

Our *in vitro* results support the conclusion that constitutive endogenous production of cobalamin compels M. smegmatis to rely on MetH for the biosynthesis of methionine. We found that the disruption of MetH activity retarded growth in the presence of cobalamin, ostensibly owing to methionine depletion. In contrast, in the absence of cobalamin, bacilli were relieved of riboswitch-mediated repression of MetE, allowing the alternative methionine synthase to substitute for the inactivated MetH. We predict that *metH* will be essential in all mycobacteria capable of *de novo* cobalamin biosynthesis, representing an important deviation from cobalamin-deficient pathogenic mycobacteria like M. tuberculosis. The corollary is that mycobacterial species that are incapable of *de novo* cobalamin biosynthesis will accommodate MetH inactivation. Indeed, *metH*-null M. tuberculosis mutants have been generated (18) and several naturally occurring, potentially inactivating mutations in *metH* have been found in circulating M. tuberculosis clinical isolates (13). Consistent with our findings, a recent Tn-screen identified *metH* among the subset of genes essential for the growth of M. smegmatis *in vitro* (27). Another recent study reported the inactivation of MetH in M. smegmatis, but in contrast to our findings, the authors did not observe any growth inhibition in their *metH*-null mutants in supplement-free media (36). The results presented here, together with our own independent Tn-seq and CRISPRi-seq analyses of M. smegmatis gene essentiality (28), demonstrate that *metH* cannot be disrupted in a cobalamin-replete strain without sacrificing viability. This apparent conflict might be explained by the possibility that the *metE* riboswitch in the parental strains used by Guzzo et al. to generate their *metH*-null mutants harbored inactivating mutations, which can accumulate spontaneously during the serial passage of mycobacterial cultures (51, 52). To eliminate the potential confounding effect of mutations in our study, the wild-type strain and its derivative mutant strains were subjected to whole-genome sequencing.

In summary, we have shown that M. smegmatis, a nonpathogenic mycobacterium, is a constitutive producer of cobalamin *in vitro*. Surprisingly, the transport of corrinoids in M. smegmatis appears restricted despite the presence in the genome of multiple putative transporters. Notably, this study also revealed differences in the regulation of methionine biosynthesis between M. smegmatis and M. tuberculosis. These differences in cobalamin-dependent metabolism between an environmental mycobacterium and an obligate pathogen might be informative in understanding the selective pressures that have shaped M. tuberculosis metabolism for pathogenicity and host tropism.

## MATERIALS AND METHODS

### Bacterial strains and culture conditions.

The bacterial strains and plasmids used in this study are described in Table S1 in the supplemental material. Unless specified, M. smegmatis cultures were grown in either Middlebrook (Difco) 7H9 broth supplemented with 10% oleic acid-albumin-dextrose-catalase (OADC) (Becton, Dickinson) and 0.05% Tween 80 or on Middlebrook (Difco) 7H10 agar supplemented with 10% OADC. For mycobacterial cultures, kanamycin (Km) and hygromycin (Hyg) were used at final concentrations of 25 μg/ml and 50 μg/ml, respectively. Escherichia coli was cultured in LB or LA with 50 μg/ml Km or 200 μg/ml Hyg, where appropriate. All cultures were incubated at 37°C. To generate growth curves, 50 μl of M. smegmatis cells were seeded at a concentration of 1 × 10^6^ CFU/ml in 96-well culture plates (Greiner Bio-One) and absorbance measurements were recorded every 1.5 h, over a period of 30 h, in a FLUOstar OPTIMA microplate reader (BMG Labtech).

### Cloning.

The oligonucleotides used for cloning and PCR are listed in Table S2. An in-frame, unmarked deletion in M. smegmatis *cobK* (*MSMEG_3875*) was generated by joining a 912-bp PCR-generated fragment (FR1) containing 40 bp of the 5′ end of *cobK* to a second 923-bp PCR-generated fragment (FR2) containing 107 bp of the 3′ end of *cobK* in a three-way ligation reaction with a p2NIL backbone (Addgene plasmid number 20188) (53), using Asp718I, BglII, and HindIII restriction. The resultant vector (p3875K) contained a deleted 120-bp *cobK* allele. To generate an in-frame, unmarked deletion in M. smegmatis *metH* (*MSMEG_4185*), a 1,524-bp amplicon (FR1) of the 5′ coding sequence of *metH* and another 1,480-bp amplicon (FR2) containing 354 bp of the 3′ end of *metH* were joined in a three-way ligation reaction with p2NIL using Asp718I, HindIII, and BglII to produce the p4185K vector carrying a truncated *metH* allele of 1,848 bp. Counter-selection fragments carrying the *lacZ*, *hyg*, and *sacB* genes was excised from pGOAL19 (Addgene plasmid number 20190) (53) and cloned at PacI sites of p3875K and p4185K to generate the suicide vectors p3875K19 and p4185K19, respectively. To generate the *hyg*-marked *metH* construct, a *hyg* cassette was excised from the pIJ963 vector (54) and cloned into the BglII site of p4185K. A counter-selection cassette derived from pGOAL17 (Addgene plasmid number 20189) (53) was then inserted into p4185K to generate p4185K17. Constructs were validated by restriction enzyme mapping and Sanger sequencing using the primers listed in Table S2.

### Isolation of allelic exchange mutants.

M. smegmatis Δ*cobK* and Δ*cobK* Δ*metH* mutants were generated by allelic exchange mutagenesis (26). A total of 100 μl of competent cells were incubated in a 1 mm cuvette with 1 to 8 μg DNA for 20 min on ice prior to pulsing in a GenePulser Xcell electroporator (Bio-Rad) with time constant and voltage settings at 5 ms and 1,200V, respectively. Single crossover (SCO) transformants were selected with Km and Hyg on 7H10-OADC plates. As colonies became visible, 50 μl of 2% (wt/vol) 5-bromo-4-chloro-3-indolyl-β-d-galactoside (X-gal) was underlain in each plate for blue/white screening of SCOs. PCR-verified SCOs were then cultured in antibiotic-free 7H9-OADC, followed by 10-fold serial dilutions and plating on 7H10-OADC containing 2% (wt/vol) sucrose. DCOs were screened by PCR and confirmed with Southern blotting or Sanger sequencing. For Southern blotting confirmation of Δ*cobK*, 2 to 3 μg DNA was digested overnight with StyI, separated on 1% agarose gel at 80 V, transferred and fixed onto a Hydrobond N+ membrane (Amersham), and hybridized overnight at 42°C with target-specific PCR-generated probes labeled with the ECL direct nucleic acid labeling and detection systems (Amersham). The target DNA fragments were visualized on Kodak hypersensitive X-ray films.

### Cobalamin extraction.

Wild-type or mutant M. smegmatis strains were cultured until stationary phase (OD_600_, ∼2) in 50 ml 7H9-OADC supplemented with 3 μg/ml cobalt chloride. Cells were harvested by centrifugation at 4,000 × *g* for 10 min at 4°C, resuspended in 8 ml of 50 mM sodium acetate buffer (pH 4.5), and stored at –80°C until needed. Once thawed, the cells were lysed by 5 min of sonication using a microtip sonicator set at 30 amplitude, 15 s pulse on and 15 s pulse off. Next, 16 μl of 100 mM KCN was added to the lysed cells and, with the extraction tube tightly closed, the samples were incubated at room temperature for 30 min in a chemical fume hood, followed by boiling at 90°C for 45 min inside the hood. The tube was then cooled on ice briefly and centrifuged at 4°C at 4,000 × *g* for 10 min. The supernatant was filtered through a 0.22-μm filter and loaded onto a Sep-Pak C18 Plus light cartridge (Waters) which had been washed with 5 ml 75% (vol/vol) ethanol and conditioned with 10 ml of sterile water. Next, the cartridge was washed with 10 ml of water and eluted with 75% ethanol, collecting about 15 drops. The eluent was analyzed immediately by LC-MS/MS or stored in –20°C until needed. When analysis was done on frozen samples, a centrifugation at 14,000 × *g* for 10 min on a benchtop centrifuge was first performed, followed by chloroform purification.

### LC-MS/MS detection and analysis of cobalamin.

Eluents were analyzed using light chromatography-tandem mass spectrometry (LC-MS/MS) in a positive ionization mode and quantitated using the following multiple reaction monitoring (MRM) parameters: *m/z*, 678→359 and *m/z*, 678→147. Chromatographic separation was performed through a high-performance liquid chromatography (HPLC) reverse-phase column (Phenomenex Synergi Polar-RP 100 Å, 50  by  2 mm [Separations]) using an Agilent 1200 Rapid Resolution HPLC system equipped with a binary pump, degasser, and autosampler, coupled to an AB Sciex 4000 QTRAP hybrid triple quadrupole linear ion-trap spectrometer. Mobile phases were 0.1% formic acid in water (A) and 0.1% formic acid in acetonitrile (B). The following gradients were run: 0 to 2 min, 95% A; 2 to 4 min, 5% A; 4 to 6 min, 95% A; and 6 to 8 min, 95% A at a flow rate of 400 μl/min. The mass spectrometry analysis was performed on an AB Sciex 4000 QTRAP LC mass spectrometer using the following parameters: curtain gas (25.00); IS (5,500.00); temperature (200.00°C); GS1 (80.00); GS2 (55.00); EP (12.0). Data processing was done using the SCIEX Analyst software.

### Quantitative gene expression analysis by ddPCR.

Droplet digital PCR (ddPCR) and data analysis was performed as described previously (55). Total RNA was extracted using the FastRNA Pro Blue kit (MP Biomedicals) and DNase treated with TURBO DNase (Ambion), after which 0.5 μg was used as the template for cDNA synthesis, using the High Capacity RNA to cDNA kit (Thermo Fisher Scientific). Primers and minor groove binder (MGB) TaqMan probes (Table S2) were designed using Primer Express 3.0 (Applied Biosystems). For duplexing, TaqMan MGB probes homologous to the target genes were labeled with 2′-chloro-7′-phenyl-1,4-dichloro-6-carboxyfluorescein (VIC), whereas those binding the reference gene, *sigA*, were labeled with 6-carboxyfluorescein (FAM).

### Targeted protein mass spectrometry.

Triplicate cultures of M. smegmatis were grown to an OD_600_ of ∼1.2 in 7H9-OADC with or without 10 μM CNCbl. Cell lysis, fractionation, and the generation of tryptic peptides was done as previously described (25). Selected reaction monitoring (SRM) assays were developed in Skyline (version 4.1) using a spectral library generated from previous discovery MS data (25) with a cutoff score of 0.9. Skyline was set up to select two peptides per input protein, with the highest picked MS1 intensity in the discovery data, and then the top 5 most intense fragment ions for each of those peptides. A transition list was then generated for the Thermo Scientific triple stage quadrupole (TSQ) Vantage mass spectrometer. Samples were separated using a Thermo Accella LC system on a 10-cm monolithic C_18_ column (Phenomenex) with a 4.6-mm ID with a mobile phase that comprised a mixture of solvent A (water plus 0.1% formic acid) and solvent B (HPLC-grade acetonitrile plus 0.1% formic acid). The method run time was 45 min in total with a flow rate of 300 μl/min. The gradient program began with 3% B, followed by a gradient of 8% to 45% B from 5 to 25 min, and then an increase to 80% B at the 30-min mark for a 5-min wash, before returning to 3% B for the remainder of the method. The LC system was run in-line into a Thermo TSQ Vantage through a heated electrospray ionization (HESI) source. The source voltage was +3,500 V, with a capillary temperature of 300°C, a vaporizer temperature of 200°C, sheath gas of 30, and aux gas of 10. To determine the retention time for each peptide, methods were generated for the TSQ Vantage with a maximum of 20 transitions monitored per method. Since the original list contained 5 transitions, a total of 8 unscheduled methods were generated, with a cycle time of 5 s to maximize the amount of signal, a collision gas pressure of 1.5 mTorr, a Q1 peak width (FWHM) of 0.7, and collision energies as determined by Skyline. The unscheduled methods were then run with consecutive 2-μl injections of a reference sample to further refine the list of transitions and determine the retention time for each peptide. The reference sample was generated by pooling all samples. The unscheduled runs were analyzed in Skyline to determine the retention times for each peptide. Any transitions with no intensity, background-level intensity, interference, or ambiguous signal were removed from the method, and a minimum of 3 transitions per peptide were kept in the final list. The spectral library was used to further refine the assays and any peptides with a *dotp* score lower than 0.7 were removed from the final list.

### Microplate alamarBlue assay.

Cell viability was determined using the microplate alamarBlue assay (24) as follows: 50 μl of 1:1,000-diluted exponential-phase cultures (OD_600_, ∼0.5) was added to 50 μl 7H9-OADC with or without 10 μM CNCbl in a 96-well plate. Plates were incubated overnight at 37°C, after which 10 μl of 100-μg/ml resazurin was added to each well. The plates were incubated for an additional 5 h at 37°C before fluorescence intensity measurements were taken using a FLUOstar OPTIMA microplate reader (BMG Labtech) using excitation and emission wavelengths of 485 nm and 508 nm, respectively.

### Gene silencing using CRISPRi.

Thirteen pairs of sgRNA oligonucleotides targeting the M. smegmatis *metH* ORF (Table S3) were designed as described previously (28). The oligonucleotides were annealed and cloned into the PLJR962 plasmid using BsmBI restriction sites in an overnight ligation reaction with T4 DNA ligase (NEB). Following ligation, the entire reaction mix (10 μl) was transformed into 50 μl of electrocompetent E. coli DH5α cells and selected on LB plates with 50 μg/ml Km. Plasmid DNA was extracted from single colonies and validated by Sanger sequencing using primer 1834 (Table S3). Next, competent M. smegmatis cells were transformed by electroporation with 200 ng of *metH* cKD constructs or an *mmpL3* cKD control and selected on 7H10-OADC containing 25 μg/ml Km with or without 100 ng/ml ATc.

### Whole-genome sequencing, genome assembly, and variant detection.

Genomic DNA was extracted as described by van Helden et al. (56) from exponential-phase cultures of single colonies. Genomic libraries, prepared using the TruSeq Nano DNA (Illumina) sample preparation kit according to the manufacturer’s instructions, were sequenced using a 150-bp paired-end strategy on an Illumina HiSeq 4000 instrument. Trimmomatic v0.35 (57) was used to remove adapters, leading or trailing bases with a quality score of <3, reads shorter than 36 bp in length, and bases with an average quality score of <15 based on a 4-base sliding window. BWA v0.7.12 (58, 59) was then used to map paired-end reads to the M. smegmatis mc^2^155 reference genome (GenBank accession number CP000480.1). SAMtools v0.1.2 (61) was used to call bases. Sites that had Phred scores lower than 20 or coverage below 10-fold were removed from further analysis. SNPeff v4.1 (62), using the M. smegmatis mc^2^155 (uid57701) reference, was used to annotate variant positions.

### Live-cell imaging and quantification of the growth of microcolonies.

A 100-μl bacterial suspension of 2.0 × 10^6^ cells/ml was prepared and loaded on the four-chambered CellASIC ONIX B04A-03 microfluidic platform (Merck). Cells were trapped with the following pressure and flow time settings: channel A8 at 13.8 kPa for 15 s; channel A6 at 27.6 kPa for 15 s. Channel A6 was then rinsed at 6.9 kPa for 30 s. Untrapped cells were washed out by flowing inlet solution at 34.5 kPa for 5 min. 7H9-OADC medium containing 25 μg/ml Km with or without 100 ng/ml ATc was perfused continuously for 43 h. Live-cell imaging was performed on a Zeiss AxioObserver using a 100×, 1.4-numerical-aperture (NA) objective with phase contrast and a Colibri.7 fluorescent illumination system. Images were captured every 15 min using a Zeiss Axiocam 503 and analyzed using FIJI software (https://fiji.sc/). To quantify the growth of microcolonies, a threshold for the time-lapse images was set with a Yen filter in FIJI and the thresholded area over time was then quantified. For each strain type, data extraction and all subsequent analyses were performed on four independent fields of view. The data were analyzed using “R” software. Growth curves were generated by subtracting initial background objects from the size data over time and smoothed with a loess regression. Growth rates were predicted from fitting a linear model to the log_2_ microcolony size obtained between 3 h and 18 h.

### Data availability.

Raw fastq files for whole-genome sequencing data for M. smegmatis mc^2^155, Δ*cobK*, and Δ*cobK* Δ*metH* are available in the European Nucleotide Archive under the accession numbers ERS3716042, ERS3716043, and ERS3716041, respectively. Movies S1 to S5 can be accessed at https://uct.figshare.com/s/65105b9914196c4b4654.

## Supplementary Material

Supplemental file 1
