# Supplementary material for: De Novo Cobalamin Biosynthesis, Transport, and Assimilation and Cobalamin-Mediated Regulation of Methionine Biosynthesis in Mycobacterium smegmatis
Source: J Bacteriol. 2021 Mar 8;203(7):e00620-20. doi: 10.1128/JB.00620-20 (PMC8088520; doi:10.1128/JB.00620-20)
Supplement: Supplemental file 1 [file JB.00620-20-s0001.pdf]

SUPPLEMENTAL MATERIAL

De novo cobalamin biosynthesis, transport and assimilation and cobalamin-mediated regulation of methionine biosynthesis in *Mycobacterium smegmatis*

Terry Kipkorir[1], Gabriel T. Mashabela[1#], Timothy J. De Wet[1,2], Anastasia Koch[1], Lubbe Wiesner[3], Valerie Mizrahi[1,4], Digby F. Warner[1,4*]

[1]SAMRC/NHLS/UCT Molecular Mycobacteriology Research Unit, DSI/NRF Centre of Excellence for Biomedical TB Research, Department of Pathology and Institute of Infectious Disease and Molecular Medicine, Faculty of Health Sciences, University of Cape Town, Observatory 7925, South Africa.

[2]Department of Integrative Biomedical Sciences, Faculty of Health Sciences, University of Cape Town, Observatory 7925, South Africa.

[3]Division of Clinical Pharmacology, Department of Medicine, University of Cape Town, Cape Town, South Africa.

[4]Wellcome Centre for Infectious Diseases Research in Africa, Faculty of Health Sciences, University of Cape Town, Observatory 7925, South Africa.

[#]Current address: DSI/NRF Centre of Excellence for Biomedical Tuberculosis Research, Division of Molecular Biology and Human Genetics, Department of Biomedical Sciences, Faculty of Medicine and Health Sciences, Stellenbosch University, Tygerberg 7505, SOUTH AFRICA

*Corresponding author. Email: digby.warner@uct.ac.za; Tel. +27 21 406 6556

Table S1: Strains and plasmids used in this study

Strain or plasmid	Description	References
Mycobacterium smegmatis		
**mc^{2}155**	High-frequency transformation mutant of *M. smegmatis* ATCC 607	(1)
**Δ*cobK***	*cobK* knock-out in *M. smegmatis* mc^{2}155	This study
**Δ*cobK*Δ*metH***	*metH* knock-out in Δ*cobK*	This study
**Δ*metE cobK*::*hyg***	*metE* knock-out and insertion of a *hyg* cassette in *cobK*	Dr. Stephanie Dawes, unpublished
***metH* cKD**	*M. smegmatis metH* conditional knockdown strain	This study
**Plasmids**		
**p2NIL**	Suicide plasmid; Km^R	(2)
**pGOAL17**	Counter-selection cassette plasmid; Amp^R	(2)

Strain or plasmid	Description	References
**pGOAL19**	Counter-selection cassette plasmid; Amp^R	(2)
**pIJ963**	*hyg* cassette plasmid	(3)
**p3875K**	*M. smegmatis* Δ*cobK* vector; Km^R	This study
**p3875K19**	*M. smegmatis* Δ*cobK* vector containing *Pac*I cassette from pGOAL19; Km^R, Hyg^R, Suc^S	This study
**p4185K**	*M. smegmatis* Δ*metH* vector; Km^R	This study
**p4185K19**	*M. smegmatis* Δ*metH* vector containing *Pac*I cassette from pGOAL19; Km^R, Hyg^R, Suc^S	This study
**p4185K17**	*M. smegmatis* Δ*metH*::*hyg* vector containing *PacI* cassette from pGOAL19; Km^R, Hyg^R, Suc^S	This study
**PLJR962**	CRISPRi backbone for *M. smegmatis*	(4)
**PLJR962_metH1**	*M. smegmatis metH* knock-down construct with sgRNA1 oligo	This study

Strain or plasmid	Description	References
**PLJR962_metH2**	*M. smegmatis metH* knock-down construct with sgRNA2 oligo	This study
**PLJR962_metH3**	*M. smegmatis metH* knock-down construct with sgRNA3 oligo	This study
**PLJR962_metH4**	*M. smegmatis metH* knock-down construct with sgRNA4 oligo	This study
**PLJR962_metH5**	*M. smegmatis metH* knock-down construct with sgRNA5 oligo	This study
**PLJR962_metH6**	*M. smegmatis metH* knock-down construct with sgRNA6 oligo	This study
**PLJR962_metH7**	*M. smegmatis metH* knock-down construct with sgRNA7 oligo	This study
**PLJR962_metH8**	*M. smegmatis metH* knock-down construct with sgRNA8 oligo	This study
**PLJR962_metH9**	*M. smegmatis metH* knock-down construct with sgRNA9 oligo	This study

Strain or plasmid	Description	References
**PLJR962_metH10**	*M. smegmatis metH* knock-down construct with sgRNA10 oligo	This study
**PLJR962_metH11**	*M. smegmatis metH* knock-down construct with sgRNA11 oligo	This study
**PLJR962_metH12**	*M. smegmatis metH* knock-down construct with sgRNA12 oligo	This study
**PLJR962_metH15**	*M. smegmatis metH* knock-down construct with sgRNA15 oligo	This study
**PLJR962_mmpL3**	*M. smegmatis mmpL3* knock-down construct	This study

Table S2: Oligos used for cloning, PCR screening, Sanger sequencing and gene expression analysis

Oligo ID	5' → 3' sequence	Description
**3875F1**	CTCAGAAAGCTTGAAAGGCGGCGATT	Forward primer for FR1 Δ*cobK*; *Hin*dIII
**3875R1**	GCAGCAGAACTCGCAGATCTATCGTC	Reverse primer for FR1 Δ*cobK*; *Bgl*II
**3875F2**	TGGCGGAGATCTTGATCATGGTGGAC	Forward primer for FR2 Δ*cobK*; *Bgl*II
**3875R2**	GGTCCGAGCATGCGGTACCGTTCTA	Reverse primer for FR2 Δ*cobK*; *Asp*718I
**4185F1**	GAGACGTTGGTACCGAACAT	Forward primer for FR1 Δ*metH*; *Asp*718I

Oligo ID	5’ → 3’ sequence	Description
**4185R1**	GCGCCCGCAGATCTGCTT	Reverse primer for FR1 Δ*metH*; *Bgl*II
**4185F2**	GCACCGAGATCTGGGCGT	Forward primer for FR2 Δ*metH*; *Bgl*II
**4185R2**	GGTGTCGAAGCTTACCGGA	Reverse primer for FR2 Δ*metH*; *Hin*dIII
**3875_SF1**	CTCAGAGAAAGGCGGCGAT	Flanking primers for Δ*cobK* 5’ SCO PCR screening
**3875_SR1**	GGCGACGACATGATGGT	
**3875_SF2**	GTTACCTGTACTCGGCGA	Flanking primers for Δ*cobK* 3’ SCO PCR screening
**3875_SR2**	CGGTGAGGGAGCAGATTT	
**4185_SF1**	GTTGAGCTTGTCGGCGAT	

Oligo ID	5’ → 3’ sequence	Description
**4185_SR1**	GCGGGTGCGAGAAATACCA	Flanking primers for Δ*metH* 5’ SCO PCR screening
**4185_SF2**	GATTGCACTGACGCGCTGA	Flanking primers for Δ*metH* 3’ SCO PCR screening
**4185SR2**	CGAAAAGTAATGGCGCCCA	
**3875_KO_F**	GGTGCGTATCGGAGGATT	Internal primers Δ*cobK*
**3875_KO_R**	GTTCGCCCTCGTAGTCAT	
**3875_F**	CAACATCGTCCCCCAACTGA	Flanking primers Δ*cobK*
**3875_R**	CGACATCACGCTCGACAAAC	
**4185-KO-F**	GTTCCTGTTCCACGCCAT	Internal primers Δ*metH*
**4185-KO-R**	CTGTCAGCCACTTCTCCT	
**4185_F**	CAACATGGACGAGGGCATGA	Flanking primers Δ*metH*
**4185_R**	GACTGCGGGTGCGAGAAATA	

Oligo ID	5’ → 3’ sequence	Description
**6638_F**	GGTTCATCGCCTCGTGGAAT	Flanking primers Δ*metE*
**6638_R**	GTCAACTTGTCAGGGCTGCT	
**3875_ProbeF**	GGGTGCACAGCGTCACCA	Primers generating 562bp probe for southern blot on Δ*cobK*
**3875_ProbeR**	CCGACATCACGCTCGACA	
**rt_metE_Fwd**	GGAGCGCAACGACATGGT	Sequence detection primers for *metE* gene expression by ddPCR
**rt_metE_Rev**	TCTCGGTCGCGAAGAAACC	
**rt_sigA_Fwd**	GCCCGCACCATCCGTAT	Sequence detection primer for *sigA* gene expression by ddPCR
**rt_sigA_Rev**	ATACGGCCGAGCTTGTTGAT	

Oligo ID	5’ → 3’ sequence	Description
**rt_metE_probe**	TATTTCGCCGAACAGC	Taqman hydrolysis probe for *metE*
**rt_sigA_probe**	CCGGTGCACATGGT	Taqman hydrolysis probe for *SigA*
**MSMcobK_SeqKF1**	CAAGGCATGACGGTCTAC	Sanger sequencing primer
**MSMcobK_SeqKF2**	ACGCCAAGGTGATCGACA	Sanger sequencing primer
**MSMcobK_SeqKF3**	CAACATCGTCCCCCAACT	Sanger sequencing primer
**MSMcobK_SeqKF4**	GGGGTCGAAGAGCATGTT	Sanger sequencing primer

Oligo ID	5' → 3' sequence	Description
**MSMcobK_SeqKR1**	CGCCGAGTACAGGTAACT	Sanger sequencing primer
**MSMcobK_SeqKR2**	ATGATGGTGGTGATGGCG	Sanger sequencing primer
**MSMcobK_SeqKR3**	GAGACCTGTTGGCGCGAT	Sanger sequencing primer
**MSMcobK_SeqKR4**	CGACATCACGCTCGACAA	Sanger sequencing primer
**MSMcobK_SeqKR5**	CGGTGAGGGAGCAGATTT	Sanger sequencing primer
**MSMmetHKO_SeqF1**	CGATCGTTGTCAGAAGTA	Sanger sequencing primer

Oligo ID	5’ → 3’ sequence	Description
**MSMmetHKO_SeqR1**	GAAACAGCCCACCGGATA	Sanger sequencing primer
**MSMmetHKO_SeqF2**	GCAGGAGAGTGATACCGAT	Sanger sequencing primer
**MSMmetHKO_SeqR2**	GTCGACTCCGAAAAGCTGT	Sanger sequencing primer
**MSMmetHKO_SeqF3**	CCCGACGCTTTAGTCACA	Sanger sequencing primer
**MSMmetHKO_SeqR3**	CAGTACTTCGTCGTCGGCA	Sanger sequencing primer
**MSMmetHKO_SeqF4**	CTTGAACAACGTGGCCTT	Sanger sequencing primer

Oligo ID	5’ → 3’ sequence	Description
**MSMmetHKO_SeqR4**	CAAGGTCGTGGAGGGCAAA	Sanger sequencing primer
**MSMmetHKO_SeqF5**	CGATCCGGTGATGTTGGT	Sanger sequencing primer
**MSMmetHKO_SeqR5**	GAATACAGCACCCCGGACA	Sanger sequencing primer
**MSMmetHKO_SeqF6**	GTTGAGGGTGTCGAAGAT	Sanger sequencing primer
**MSMmetHKO_SeqF7**	GGGTTGAGTGTTGTTCCA	Sanger sequencing primer
**MSMmetHKO_SeqR6**	GGGTTCCGATTTAGTGCT	Sanger sequencing primer

Table S3: sgRNAs targeting *M. smegmatis metH* gene

sgRNA ID	Distance from *metH* TSS	Target complementarity score (4)	Oligo	5' → 3' Sequence
**sgRNA1**	3603	3	F1	GGGAAAGTACTGCGACTGCGGGTG
			R1	AAACCACCCGCAGTCGCAGTACTT
**sgRNA2**	3060	4	F2	GGGAGTCGTCACCGACGGCGTTGGC
			R2	AAACGCCAACGCCGTCGGTGACGAC
**sgRNA3**	3057	1	F3	GGGAGTCACCGACGGCGTTGGCCGG
			R3	AAACCCGGCCAACGCCGTCGGTGAC
**sgRNA4**	2934	11	F4	GGGAGCCGGGTTGTTGAGGATGTC

sgRNA ID	Distance from *metH* TSS	Target complementarity score (4)	Oligo	5' → 3' Sequence
			R4	AAACGACATCCTCAACAACCCGGC
**sgRNA5**	2904	5	F5	GGGAGCCCTTCATCTCCCACGCGTT
			R5	AAACAACGCGTGGGAGATGAAGGGC
**sgRNA6**	2242	4	F6	GGGAGCTTCTTCTCGGCCTCGATGT
			R6	AAACACATCGAGGCCGAGAAGAAGC
**sgRNA7**	2184	11	F7	GGGAGCGGACTTCACGACCTGCGG
			R7	AAACCCGCAGGTCGTGAAGTCCGC

sgRNA ID	Distance from *metH* TSS	Target complementarity score (4)	Oligo	5' → 3' Sequence
**sgRNA8**	2002	4	F8	GGGAGTGCGTGATGCGTTCGCGCA
			R8	AAACTGCGCGAACGCATCACGCAC
**sgRNA9**	1791	11	F9	GGGAGTCCAGGCCGGCCTTGATGGC
			R9	AAACGCCATCAAGGCCGGCCTGGAC
**sgRNA10**	1785	11	F10	GGGAGCCGGCCTTGATGGCGTGGAA
			R10	AAACTTCCACGCCATCAAGGCCGGC
**sgRNA11**	1743	11	F11	GGGAGCCTCCCGCACCGGGTTGTTGCC

**sgRNA ID**	**Distance from *metH* TSS**	**Target complementarity score** (4)	**Oligo**	**5' → 3' Sequence**
			R11	AAACGGCAACAACCCGGTGCGGGAGGC
**sgRNA12**	1737	5	F12	GGGAACCGGGTTGTTGCCGCGGAA
			R12	AAACTTCCGCGGCAACAACCCGGT
**sgRNA15**	729	5	F15	GGGAGCTGGGCGTGCCGGATCGAGTT
			R15	AAACAACTCGATCCGGCACGCCCAGC
**mmpL3**			L3F	GGGAACAGACTGGCTGCCCTCGTC
			L3R	AAACGACGAGGGCAGCCAGTCTGT

sgRNA ID	Distance from *metH* TSS	Target complementarity score (4)	Oligo	5' → 3' Sequence
**P1834**				TTCCTGTGAAGAGCCATTGATAATG

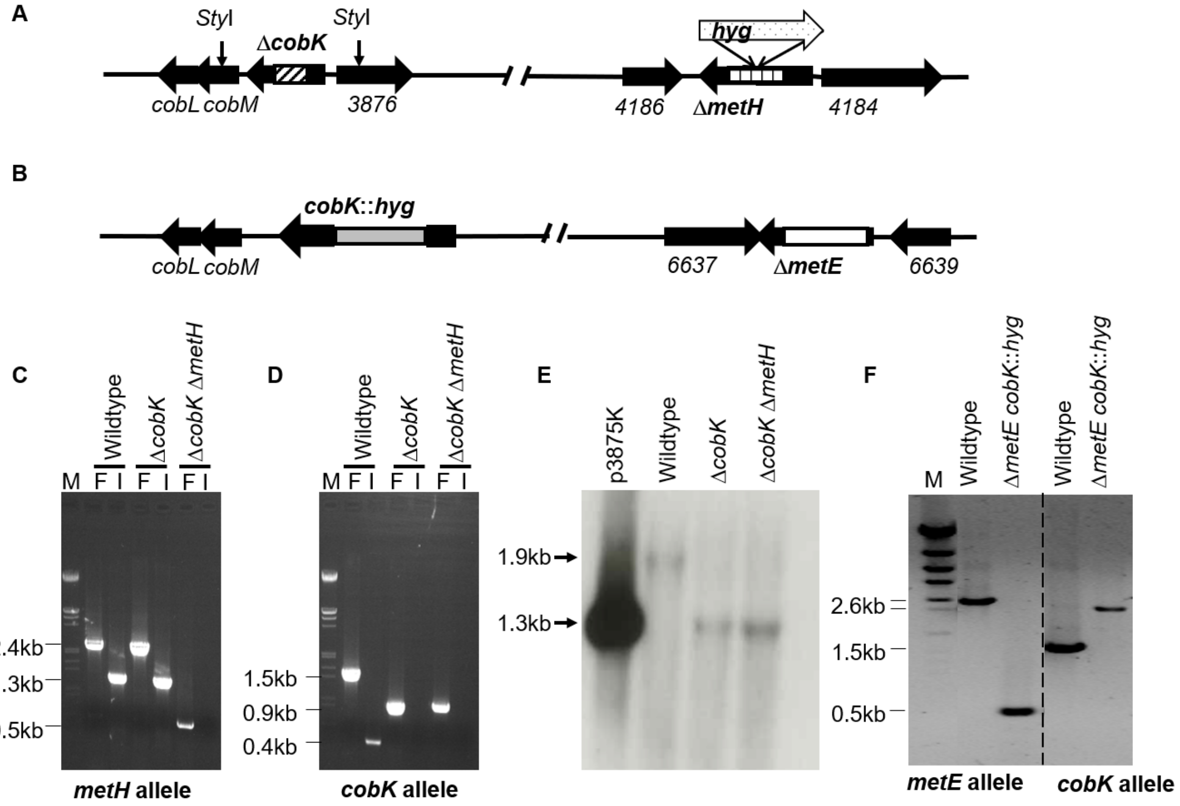

Figure S1. Construction and screening of *M. smegmatis* mutants. A. Schematic of the Δ*cobK* and Δ*metH* genotypes. The marked Δ*metH*::*hyg* construct was generated by inserting a *hyg* fragment (broad arrow with dotted pattern) into the Δ*metH* construct using *Bgl*II sites. **B.** Schematic of the Δ*metE cobK*::*hyg* genotype showing the deleted portion of the *metE* allele (white rectangle) and the insertion of a *hyg* fragment (grey rectangle) into the *cobK* allele. **C-D.** PCR screening of the putative Δ*cobK* and Δ*metH* strains using primers targeting flanking (F) or internal (I) regions of the deleted portion of each gene. Amplicon sizes for flanking primers: wild-type *metH* – 2.4kb; Δ*metH* – 0.5kb; wild-type *cobK* – 1.5kb; Δ*cobK* – 0.9kb. Amplicon sizes for internal primers: wild-type *metH* – 1.3kb; wild-type *cobK* – 0.4kb. M – DNA molecular weight marker. **E**. Confirmation of Δ*cobK* by Southern blotting. A PCR-generated probe for *cobK* was used to detect a fragment between two naturally occurring *Sty*I restriction sites (down-facing arrows in **A**). Expected fragments: wild-type *cobK* – 1.9kb; Δ*cobK* – 1.3kb. **F.** PCR genotyping of Δ*metE cobK*::*hyg* strain using primers flanking *cobK* and *metE*. Dashed line separates two independent gels.

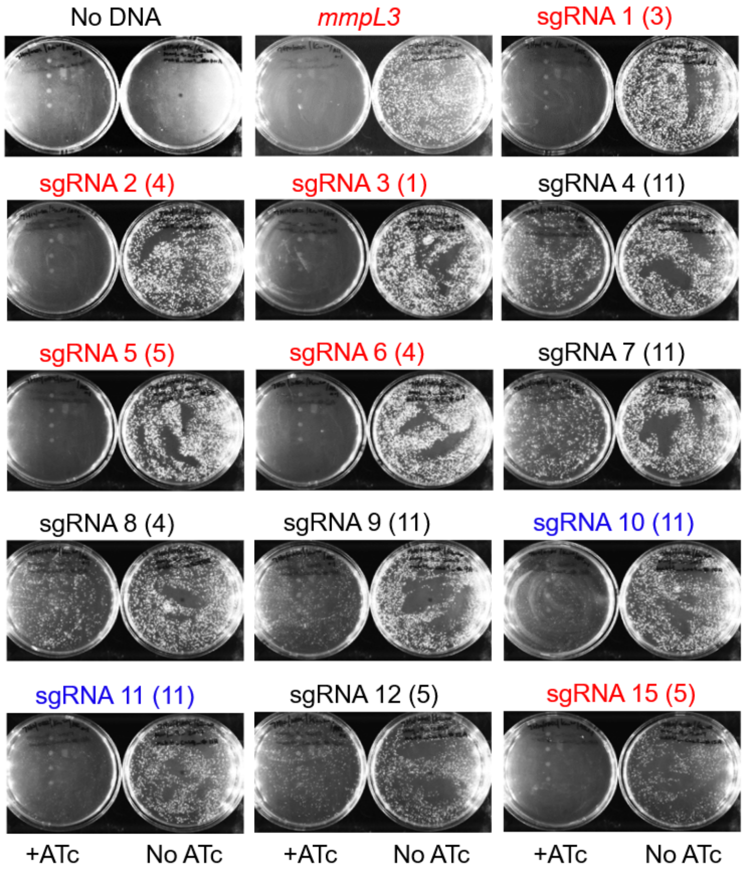

Figure S2. Conditional knockdown of *metH* using CRISPRi. The knock-down of *mmpL3* completely inhibited mycobacterial growth, whereas the 13 *metH* cKD constructs suppressed mycobacterial growth to varying degrees. The sgRNAs that produced complete inhibition of growth upon ATc addition are highlighted in red, those exhibiting partial inhibition in blue, and those with no inhibition in black. The target complementarity scores associated with each sgRNA are indicated in parentheses.

Supplementary Movies

Supplementary Movies S1–5 can be accessed at https://uct.figshare.com/s/65105b9914196c4b4654. Live-cell imaging of wild-type *M. smegmatis* mc^{2}155 and *metH* cKD strains using time-lapse, phase-contrast microscopy. For single-cell analysis using microfluidics, a suspension of 2 ×10^6 bacterial cells/mL at exponential growth phase was preincubated with or without 100ng/mL of ATc for 6 h at 37°C prior to loading on the microfluidics platform. The experiment was run for 43 h and images were captured every 15 min.

REFERENCES

1. Snapper SB, Melton RE, Mustafa S, Kieser T, Jr WRJ. 1990. Isolation and characterization of efficient plasmid transformation mutants of Mycobacterium smegmatis. Mol Microbiol 4:1911–1919.

2. Parish T, Stoker NG. 2000. Use of flexible cassette method to generate a double unmarked Mycobacterium tuberculosis tlyA plcABC mutant by gene replacement. Microbiology 146:1969–1975.

3. Blondelet-Rouault M-H, Weiser J, Lebrihi A, Branny P, Pernodet J-L. 1997. Antibiotic resistance gene cassettes derived from the Ω interposon for use in E. coli and Streptomyces. Gene 190:315–317.

4. Rock JM, Hopkins FF, Chavez A, Diallo M, Chase MR, Gerrick ER, Pritchard JR, Church GM, Rubin EJ, Sassetti CM, Schnappinger D, Fortune SM. 2017. Programmable transcriptional repression in mycobacteria using an orthogonal CRISPR interference platform. Nat Microbiol 2.
